# 

**Published:** 1904-10

**Authors:** 


**This Journal and Atlanta Semiweekly Journal one year for $1.25 cash in advance. No checks. Strictly to new subscribers.**

### BOOK REVIEWS.

### Books Received.

Rapiotherapy, Phototherapy and High Frequency Currents. The Medical and Surgical Applications of Radiology in Diagnosis and Treatment. By Charles Warrenne Allen, M.D., Professor of Dermatology in the New York Post-Gradnate Medical School. Octavo, 618 pages, 131 engravings and 27 plates. Price, cloth, $4.50, net. Philadelphia and New York: Lea Bros. and Co., Publishers.

A Practical Treatise on Genito-Urinary and Venereal Diseases and Syphilis. By Robert W. Taylor, A.M., M.D. Third edition, thoroughly revised. 163 illustrations and 39 plates in colors and monochrome. New York and Philadelpnia: Lea Bros. & Co.

A Treatise on the Principles and Practice of Gynecology. By E. C. Dudley, A.M., M.D., Professor of Gynecology in the Northwestern University Medical School, Chicago. New (4th) edition. Revised and enlarged. Octavo, 771 pages, with 401 illustrations, of which 50 are in colors and 18 full-page colored plates. Price, cloth, $5, net; leather, $6, net; half morocco, $6.50, net. New York and Philadelphia: Lea Bros. & Co.

***NOTICE.—This JOURNAL and THE INTERNATIONAL JOURNAL OF SURGERY one year for $1.00 cash, to new subscribers. Out-of-town checks not accepted unless 10c. is added for cost of collection. Strictly to new subscribers.***

---

**This Journal and Atlanta Semi-weekly Journal one year for $1.25 cash in advance. No checks. Strictly to new subscribers.**

**NOTICE.—This JOURNAL and THE INTERNATIONAL JOURNAL OF SURGERY one year for $1.00 cash, to new subscribers. No out-of-town Checks accepted unless 10c. is added for cost of cashing.**

**This Journal and Atlanta Semi-weekly Journal one year for $1.25, cash in advance. No checks. Strictly to new subscribers.**

